# Transverse colonic volvulus after resection of sigmoid volvulus: Presentation of a case report

**DOI:** 10.1016/j.ijscr.2019.06.008

**Published:** 2019-06-12

**Authors:** Samaual Eldirdiri, Idriss H. Musa, Hawa Y. Adam, Afraa G. Suliman, Muaz M. Ata Elmanan, Sami Eldirdiri, Yasir O.M. Awadelseed, Majdi O.A. Bakhiet

**Affiliations:** a Gadarif Teaching Hospital, Gadarif, Sudan; b Sudan Medical Specialization Board, Khartoum Sudan; c Gadarif University, Sudan

**Keywords:** Gangrenous, transverse colonic volvulus, Sigmoid volvulus, Case report

## Abstract

**Introduction:**

Colonic volvulus is the third leading cause of colonic obstruction in the world following colorectal cancer and complicated sigmoid diverticulitis. Transverse colon is the rarest among other colonic volvulus. Moreover synchronous/metachronous transverse colonic volvulus is extremely rare condition with only few case reported in the literature, we hereby report this case of metachronous transverse colonic volvulus years after sigmoidectomy for sigmoid volvulus

**Case presentation:**

A case of transverse colon volvulus in elderly male with history of chronic constipation and a previous history of a sigmoid volvulus resection few years ago. He presented as an emergency with typical features of acute large bowel obstruction. He was successfully managed with a good out come

**Discussion:**

We discuss the presentation, diagnosis and surgical management with a literature review of this case of a metachronous transverse colonic volvulus.

**Conclusion:**

Metachronous colonic volvulus is an extremely rare cause of large bowel obstruction and should be considered as a differential diagnosis especially in geographic areas with high rate of volvulus or in those with underlying risk factor

## Highlights

• Volvulus is a common cause of large bowel obstruction but its metachronous occurrence is an extreme rarity.
• Metachronous transverse colonic volvulus should be eliminated as a differential diagnosis.
• Gangrenous bowel is a life threatening condition and should be dealt with as an emergency situation with the adequate procedure.
• The elective surgical resection should follow optimization of the general condition and set-up.

## 1 Introduction

Volvulus is rotation or twisting of the intestine around their vascular pedicle [1]. Colonic volvulus is the third leading cause of colonic obstruction in the world following colorectal cancer and complicated sigmoid diverticulitis [2]. According to various series in the literature, the sigmoid was involved in 60–70% of cases, cecum in 25–40% of cases, and transverse colon in 1–4% [3]. Most surgeon haven’t come across a single case of transverse colonic Volvulus, so they tend not to consider it in the deferential diagnosis of large bowel obstruction, especially if the patient had a history of previous resected volvulus. We hereby describe a rare case of a transverse colon Volvulus occurring in patient with previous history of sigmoid resection due to sigmoid volvulus. A scenario which is described in the literature once. This work has been reported in line with the SCARE criteria [4].

## 2 Case presentation

**Fig. 1:**
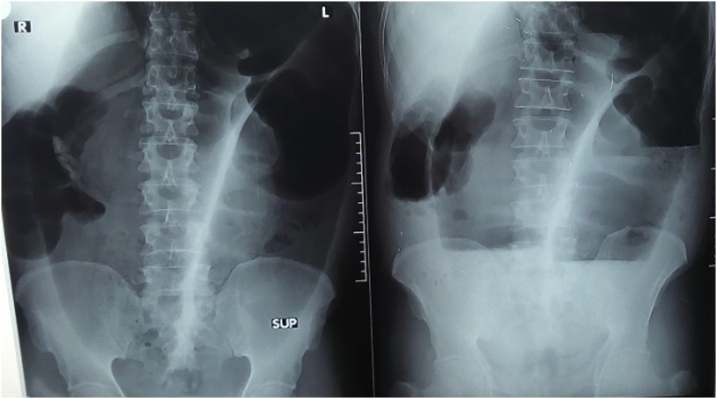
Erect and supine plain abdominal X-rays.

Sixty years old male, farmer from Gadarif city, Eastern Sudan, who had a 4 years past history of intestinal obstruction due to sigmoid volvulus for which he underwent laparotomy and detwesting sigmoidopexy followed 6 weeks later by elective sigmoidectomy. He had no significant history till 2 months ago when he presented to the surgical outpatient of Gadarif Teaching Hospital, which is the main community hospital in the state, with a history of abdominal distension, constipation and vomiting for the last 3 days and severe abdominal pain for one day. He also complained of recurrent constipation which he didn’t bother to seek medical advice and used to have over the counter or traditional medications. He was previously healthy with no significant family, drug or social history. On examination he looks ill not pale, jaundiced or febrile. His pulse rate was 104 beat per minute, blood pressure was 110/70, respiratory rate was 22 cycle per minute and his temperature was 38.8 CO. His abdomen was grossly distended with full flanks, midline scar, visible dilated bowl loop and peristalsis. There was tenderness all over the abdomen and no bowl sounds were detected. Digital rectal examination revealed a 3rd degree pile and empty rectum. His investigations showed an Hb of 13 g/dl, WBCs of 14 × 109 per liter and PLTs count of 305 × 109 per liter. His renal profile and serum electrolytes were within normal range. Blood glucose was 193 mg/dl. Urine examination was unremarkable. Abdominal X-ray (Fig. 1) showed a typically dilated omega shape colon. The diagnosis of a strangulated bowel obstruction was considered, adhesive type was the top differential. However recurrent or other site volvulus was a remote possibility despite the X-ray findings.

**Fig. 2:**
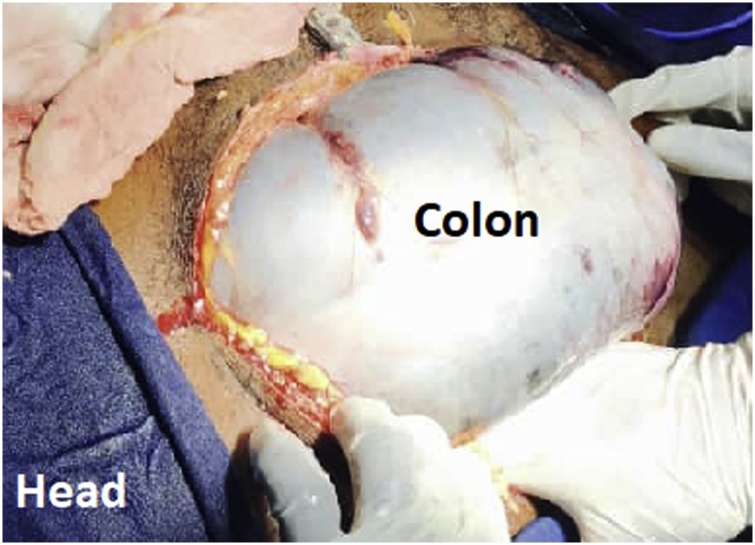
Appearance at Laparotomy.

**Fig. 3:**
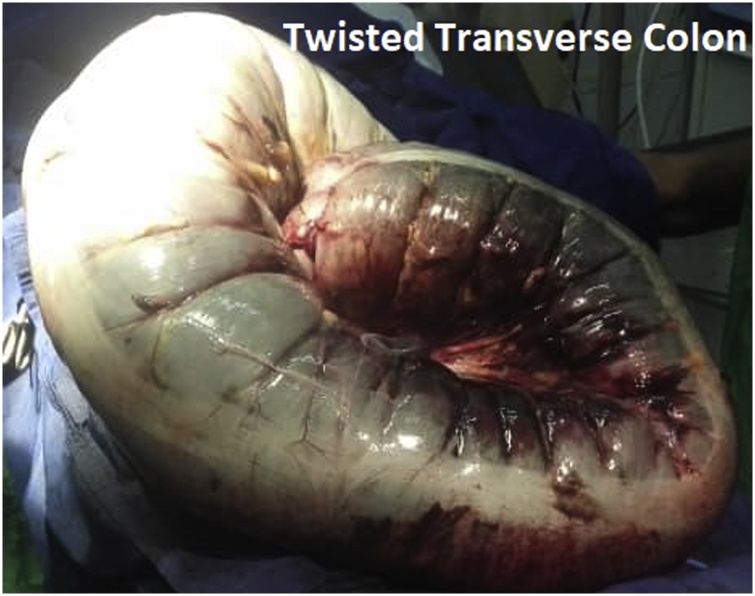
Gangrenous transverse colonic segment delivered.

**Fig. 4:**
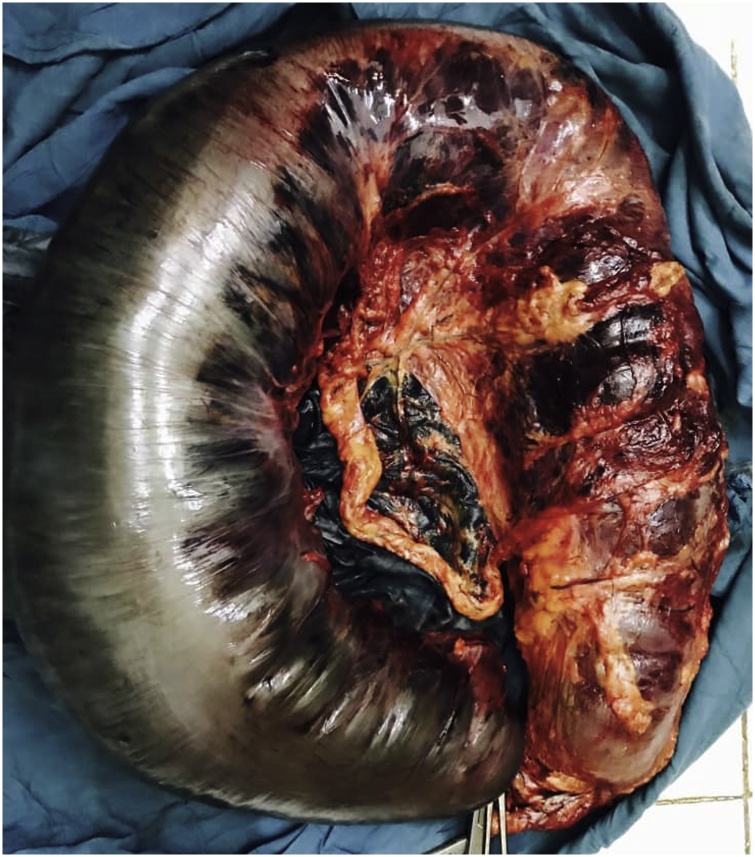
Resected Transverse Colon.

After optimization of his general condition with an NG tube suction and intravenous fluids resuscitation, he went for a laparotomy at the emergency department OR. This was performed by the specialist and residents on duty supervised by the consultant in our team. Under general anesthesia with muscle relaxation a generous midline incision revealed an intact, grossly dilated and apparently gangrenous large bowel that turned out to be the transverse colon (Fig. 2). There was a small amount of inflammatory exudate. The twisted gangrenous colon was carefully delivered (Fig. 3). Because of the high risk situation the decision was to do a Hartmann’s resection of transverse colon. A clearly viable segment was left from hepatic flexure and the healthy upper rectum which was anchored as stump to anterior abdominal wall with a nylon stitches. Abdomen closed in layers and the patient recovered smoothly. The resected colonic segment was 109 cm in length and 22 cm in its maximum diameter (Fig. 4). The patient did well postoperatively with close follow up by the nursing staff and doctors. He stayed for couple of days in the HDU and was discharged home on day 12 after healing of a minor surgical site infection. Six weeks later a colorectal anastomosis was done by our colorectal surgeon and he was discharged on the 7th post-operative day in a good condition. He presented to the refer clinic one month later completely satisfied complaining only of an unusual soft stool and regained his full normal activity. He is planned for regular clinical follow-up which can be augmented with CT or colonoscopy.

## 3 Discussion

The combination of sigmoid and transverse colonic volvulus might be synchronous or metachronous. We report this case of metachronous transverse colonic volvulus in an elderly man, who had a history of chronic constipation, after previous sigmoidectomy for a sigmoid volvulus 4 years ago. Samuel et al in 2000 described a pediatric case with metachronous sigmoid volvulus after being operated for transverse colon volvulus [5]. Recurrent/ metachronous volvulus of transverse after sigmoid resection was reported once again in an adult patient in 2009 by Booij et al. [6], while Parish et al described the only case of coexistent-synchronous volvulus of the transverse, sigmoid and right colon [7].

The typical patient of sigmoid volvulus age is between 40–60 years old, This explained by the greater frequency of constipation in those post middle age and also possibility that gut favors volvulus requires some years for preparation in contrast to transverse colonic volvulus which occur in younger, often in the second and third decade of life, and more likely female [8]. This was observed in our patient who had his sigmoid volvulus treated surgically around the age of 55 years.

Only 3–5% of all cases of intestinal obstruction are caused by colonic volvulus [9]. Among these, transverse colon is involved in 2–4 % versus 43–80% and 15–43% respectively for sigmoid colon and caecum [5,10].

Predisposing factors are congenital, physiological and mechanical [9,11]. The two congenital that causes volvulus are redundancy and non-fixation [12]. Physiological causes include high roughage diet and large bowl distension secondary to chronic constipation and associated with psychiatric and neurologic diseases [6]. Mechanical causes are previous volvulus of transverse or sigmoid colon, distal colonic obstruction, adhesion, malposition of colon fallowing previous surgery, mobility of right colon, inflammatory strictures and carcinoma [9,11]. Other reported causes are chilaiditis syndrome [11]. *Clostridium difficile* pseudo membranous colitis [13].

There is two type of presentation of volvulus, acute fulminating transverse volvulus, present with marked leukocytosis, acute abdominal pain with rebound tenderness, nausea and vomiting, but limited abdominal distension [14], and this need immediate surgical intervention in order to resect compromised bowl before gangrene or perforation occurs [11]. Other type the sub-acute progressive transverse volvulus which is associated with massive abdominal distension, mild abdominal pain without rebound tenderness and little nausea or vomiting [14]. The leukocytes count is often normal or mildly elevated, attributed to the lack of ischemia at early stages [13].

This progressive onset of symptoms can delay the diagnosis and treatment which may result in acute fulminating type with bowl infraction, peritonitis and even death [11]. Our case seems to belong to the second type, as he presents with sub-acute transverse volvulus, marked abdominal distension without rebound tenderness and decrease in bowl sound with leukocytosis, which render him going towards fulminate type at time of admission. Abdominal X-ray show dilated large bowl that raised the possibility of volvulus despite the history of previous of sigmoid resection. CT was not done due to lack of availability and the urgency of the case at the time of presentation.

Sigmoid volvulus can be decompressed by colonoscopy if presented early and there is no suspicion of strangulation, nevertheless transverse colon volvulus must be surgically treated as an emergency. Resection is treatment of choice to prevent recurrence. In fact, detorsion alone or associated colopexy has higher rate of recurrence than resection [9,11]. However, some authors recommended considering a subtotal colectomy in the presence of mega colon, instead of partial resection of the involved bowl segment [6].

Residing in the “Volvulus Belt” of Africa, in addition to the other predisposing factors described, this case completely fits within the risk profile. The colon was diffusely dilated and freely mobile suggesting that a tension-free anastomosis was achieved by mobilization of the splenic flexure in the first operation. The presence of a megacolon during primary intervention is a well-known risk factor for recurrence of volvulus and dividing the attachments of the remaining colon theoretically further increases this risk. Therefore, it is recommended to consider a subtotal colectomy in the presence of a megacolon, instead of partial resection of the involved bowel segment. [15]

## 4 Conclusion

Metachronous colonic volvulus is an extremely rare cause of large bowel obstruction that should not be eliminated as a differential diagnosis especially in those with underlying risk factors or residing in a geographic areas known with high rates of volvulus.

## Declaration of Competing Interest

Nothing to disclose.

## Funding

No funding.

## Ethical approval

Ethical approval obtained from: Gadarif University – Scientific Research Ethical Committee.

## Consent

Written and signed consent is obtained.

## Author contribution

1- Sami Eldirdiri MD, FACS: Consultant General Surgeon the main author.

2- Samaual Eldirdiri MD: Specialist General Surgeon who did the emergency operation.

3- Yasir O. M. Awadelseed MD, Clinical Fellowship: Colorectal Surgeon who did the elective reversal of Hartman’s.

4- Majdi A. O. Bakhiet MD: Specialist General Surgeon who did the previous sigmoidectomy.

5- Idriss H Musa MBBS: Resident of General Surgery: data collection and writing.

6- Hawa Y. Adam MBBS, MRCS: Resident of General Surgery: data collection and writing.

7- Afraa G. Suliman MBBS: Resident of General Surgery: data collection and writing.

8- Muaz M. Ata-elmanan MBBS: Resident of General Surgery: data collection and writing.

## Registration of research studies

This is a case report.

## Guarantor

Sami Eldiridiri.

## Provenance and peer review

Not commissioned, externally peer-reviewed.
